# Zoonoses in the margins: environmental displacement and health outcomes in the Indus Delta

**DOI:** 10.1186/s12939-022-01823-0

**Published:** 2022-12-30

**Authors:** Dorien H. Braam

**Affiliations:** Disease Dynamics Unit, Department of Veterinary Medicine, University of Cambridge, Madingley Road, CB3 0ES Cambridge, UK

**Keywords:** Disaster displacement, Climate change, Zoonotic disease, Animal health, Determinants of health

## Abstract

**Background:**

It remains unclear how human and animal displacement impacts zoonotic disease risk with little contextualized primary data available. This study investigates zoonotic disease dynamics in populations regularly displaced due to slow onset disasters and annual monsoons in the Indus Delta in Sindh province in southeast Pakistan.

**Methods:**

Using a case study methodology, semi-structured key informant interviews and focus group discussions with 35 participants, as well as observational studies were conducted in seven communities in Thatta district.

**Results:**

Key factors affecting zoonotic disease dynamics in environmental displacement in Thatta identified in the study include disasters and loss of forage, a lack of veterinary and healthcare access, and socio-economic status. Animal and human health are an important consideration in displacement disrupting communities and livelihoods, affecting safety, health, and food security. Displacement results in a poverty spiral whereby the displaced find themselves at continuous peril from poverty and disaster, with zoonotic disease dynamics shifting based on environmental changes, and an expectation of future movement and loss.

**Conclusion:**

The precarious conditions generated through the disruption of communities and livelihoods makes environmentally displaced populations vulnerable to zoonotic disease. To prevent further displacement and disease, broader political economy issues need to be addressed, and comprehensive assistance provided to support sustainable livelihoods.

**Supplementary Information:**

The online version contains supplementary material available at 10.1186/s12939-022-01823-0.

## Background

Zoonotic diseases, transmissible between animals and humans, are a significant risk to human health, as over 60 percent of infectious pathogens originates in animal species [1], with 75 percent of emerging infectious disease of zoonotic origin [2]. While diseases of zoonotic origin such as Ebola and SARS-CoV2 (COVID-19) make global headlines, endemic zoonotic diseases such as brucellosis and rabies account for an estimated 20 percent of human illness and death in low- and middle-income settings [2]. Brucellosis for instance is prevalent in cattle, goats and sheep, and may be transmitted to humans through consuming raw milk products from infected animals or via direct contact, therefore those without resources to cook, or living in unsanitary conditions close to their animals are most at risk. In Pakistan, where dairy animals are important for the livelihood of rural communities, the prevalence of brucellosis is estimated between 6–15 percent in large farms, with no data available of smallholders [3]. Meanwhile rabies, a viral transmitted to humans and other animals through the bite of a rabid dog, continues to claim up to 5,000 human deaths a year in Pakistan [4]. Lacking a comprehensive monitoring system, there are significant data gaps in disease prevalence, in particular zoonoses, with few sero-prevalence studies conducted in rural areas [5].

The risk of zoonoses depends on complex interlinked ecological, political, and socio-economic processes and conditions [6]. During disasters, disease risks are further influenced by ecological conditions, malnutrition, and overcrowding [7]. Subsequent displacement poses a challenge to the prevention and control of zoonotic disease [8], and is considered to increase zoonotic disease risk, as living conditions deteriorate and animals and humans move into new pathogen and vector environments, however, there is little contextualized primary data available on whether zoonotic disease burden in fact increases during displacement [9]. This study therefore aims to provide better insight in what I define as zoonotic disease ‘dynamics’, allowing for both increase and decrease in zoonotic disease vulnerabilities and risks, in displaced populations in the Indus Delta in Sindh province in southeast Pakistan.

The Indus Delta is an important ecosystem to the region, covering agricultural land, lakes, riverine- and mangrove forests [10], however, it is also highly vulnerable to the impacts of climate change. The mangrove forest tract provides fuel and forage to local communities, and acts as an important natural barrier against tidal waves and coastal erosion, however, its cover decreased from an estimated 2500 to less than 1000 km2 between 2000 and 2015 [11, 12]. The degradation of the ecosystem and loss of biodiversity poses zoonotic disease spillover risks, through the increased interaction between wildlife, domestic livestock and humans [13]. Some of this loss is attributed to overexploitation of natural resources by local communities, however, studies indicate that upstream water diversion and pollution are important causes of environmental degradation [11].

Reductions in Indus river water flow as a result from irrigation works upstream, have greatly affected Sindh’s soil fertility and decreased agricultural yield, through the loss of silt deposits, seawater intrusion and resulting flooding and salination [11, 14, 15]. Under the auspices of the World Bank, the Indus Water Treaty was signed in 1960, however, there are ongoing water disputes related to the tributaries of the Indus originating and passing through India [11]. Worse, some development projects supported by international financial institutions have caused damage by changing the quantity and courses of water flows, with irreversible detrimental impact to the environment and livelihoods of communities downstream [11].

Throughout the province, water availability is further impacted by accelerated glacier melting as a result of global warming, the quantity and intensity of storms and tropical cyclones with resulting tidal activity, longer heatwaves and unpredictable monsoon precipitation [16], and increasing salinity of soil and aquifers in coastal regions due to sea intrusion as a result of lack of freshwater flow and rising sea levels [11]. The freshwater flow downstream from the Kotri barrage near Hyderabad to the Delta decreased from 150 (185 billion m3) to less than 2 Million Acre Feet (MAF) (2.5 billion m3) annually, a reduction in silt deposits of 400 to less than 30 million tonnes per year, even though according to the interprovincial Water Accord of 1991 a water supply of at least 48 MAF (59 billion m3) was recommended [10, 17]. The loss of freshwater supply has destroyed 10% of cultivated land, and caused seawater intrusion up to 100 km inland, eroding over 486,000 hectares and inflicting financial losses of over US$ 2 billion [17]. Furthermore, as a result of upstream pollution, the ground- and surface water is unsuitable for drinking and agricultural purposes [18]. In the past decades, 60% of land in the coastal region in Sindh has been submerged by the sea, and up to 90% of land is now unsuitable for further cultivation, limiting livelihood and vocational opportunities [14, 15]. From 1989 to 2018 an average of 20 m of land were lost annually in eastern Sindh, while sea level rose with 1.1 to 1.9 mm/year [19].

The loss of land and livelihoods is estimated to have displaced hundreds of thousands of people in the Delta, although neither exact nor recent data is available [17]. In 2020 alone, disasters displaced almost 830,000 people in Pakistan, with thousands displaced in Sindh as a result of flooding during the monsoon season, which has become increasingly less predictable due to climate change [20]. What these data do not count, are those displaced annually during the monsoon rains, which are considered ‘regular’ seasonal migrants, even though the repeated destruction of their homes and agricultural land due to flooding can be as debilitating to their lives and livelihoods as those considered ‘displaced’ as a result of more irregular disasters. Statistics also exclude those who are affected by slow-onset disasters such as gradual coastal erosion, moving over time independently, or adapting their migration and settlement pattern according to severe weather events. Perennial agriculture and fishing livelihoods are increasingly lost to environmental degradation, with increasing rural to urban migration [14]. In the remaining villages, subject to regular flooding, infrastructure, services, fresh water and sanitation are largely absent, affecting both human and animal health.

According to the United Nations Guiding Principles on Internal Displacement, Internally Displaced Persons (IDPs) are ‘persons or groups of persons who have been forced or obliged to flee or to leave their homes or places of habitual residence (…)’, while the International Organization for Migration (IOM) considers environmental migrants as 'persons or groups of persons who, predominantly for reasons of sudden or progressive change in the environment that adversely affects their lives or living conditions, are obliged to leave their habitual homes, or choose to do so, either temporarily or permanently, and who move either within their country or abroad' [21]. In this paper, I use the definition of ‘environment’ to highlight the impact of not only the natural environment, but all ‘surroundings or conditions in which a person, animal, or plant lives or operate’, which in the Delta is largely a result of anthropogenic activities. Environmentally displaced in this paper are thereby all those who are no longer able to provide for their livelihoods in their usual place of habitancy, either permanently or temporarily.

### Study objectives

Little is known about the impact of environmental displacement on animal and human health in the Indus Delta, as communities are disconnected and detached from provincial centers, excluded from census and largely dependent on a localised economy [14]. The most recent livestock population census was conducted in 2006, and zoonotic disease data in Sindh is patchy, with the available data displaying large gaps over space and time [3, 22]. Without sufficient understanding of where these communities are and what they need, the government has little access to necessary data for policy development, including on livestock and health. This study therefore aims to improve knowledge of zoonotic disease dynamics during environmental displacement in the Indus Delta in Sindh.

Furthermore, it addresses a broader knowledge gap around zoonotic risk specifically during ‘regular’ seasonal migration and slow-onset displacement as a result of environmental degradation, an increasing challenge often not given the same attention as sudden-onset disaster displacement [23]. Its objectives are twofold: first to contextualize the lived experiences by the displaced populations, and second to provide data to better equip and inform policymakers and practitioners to better prepare for and prevent zoonotic disease risk during environmental displacement, which will undoubtedly increase as the Delta suffers the ongoing effects of climate change.

## Methods

### Study design

I adopted the case study methodology as the most relevant approach to answer an open research question about a complex contemporary phenomenon in a real-life context [24, 25]. I developed topic guides based on previous country experience, a scoping field visit and literature review, as the basis for qualitative semi-structured questionnaires. These were used to conduct key informant interviews, focus group discussions and observational studies, supported by secondary qualitative and quantitative data. A key characteristic of semi-structured interviews is that respondents can refocus the interview based on the areas they feel are most relevant. Using this method, insights and perspectives were obtained from individuals, households and communities often ignored in standard data collection [26]. The semi-structured questionnaires focused on demographic and socio-economic characteristics, experiences of displacement, human and animal health and health-seeking practices, and were refined iteratively during the interviews. The research tools are available as supplementary material.

### Population and location

Site selection was based on prior knowledge of Sindh, and a scoping trip in March – April 2019. Sindh province is dependent on irrigated agriculture fed by the water and sediment of the Indus river, after which the province is named [27]. Although roughly half of its population lives in rural areas, the province’s urbanisation rate is high. This can be partially attributed to rapid environmental changes, in particular a lack of fresh water in its coastal areas. Furthermore, while the Delta is home to one of the last remaining large mangrove forest tracts, its forests are rapidly being depleted, either through soil salinisation or the use of wood, although some rehabilitation is ongoing [17].

This study was conducted in Thatta district, based on its high disaster risk according to Pakistan’s National Disaster Management Authority (NDMA), including through sea intrusion and increasingly unpredictable rain patterns during monsoon season in July and August. While livestock contributes over 55 percent to Sindh’s total agriculture sector, with cattle, buffalo, goats and sheep the main animals kept [28], in Thatta up to 90% of the population depends on fishing, and only 8% on agriculture and forestry [11]. Around 75% of people is estimated to live below the poverty line [11]. Among respondents, livelihoods included subsistence agriculture on small plots of land owned (2–5 acres), fishing, and daily wage labour, diversified with livestock keeping. Wage labour includes construction or agriculture labour on the land of a Zamindar (landlord), a persistent feudal system institutionalised during colonialism [27].

**Fig. 1:**
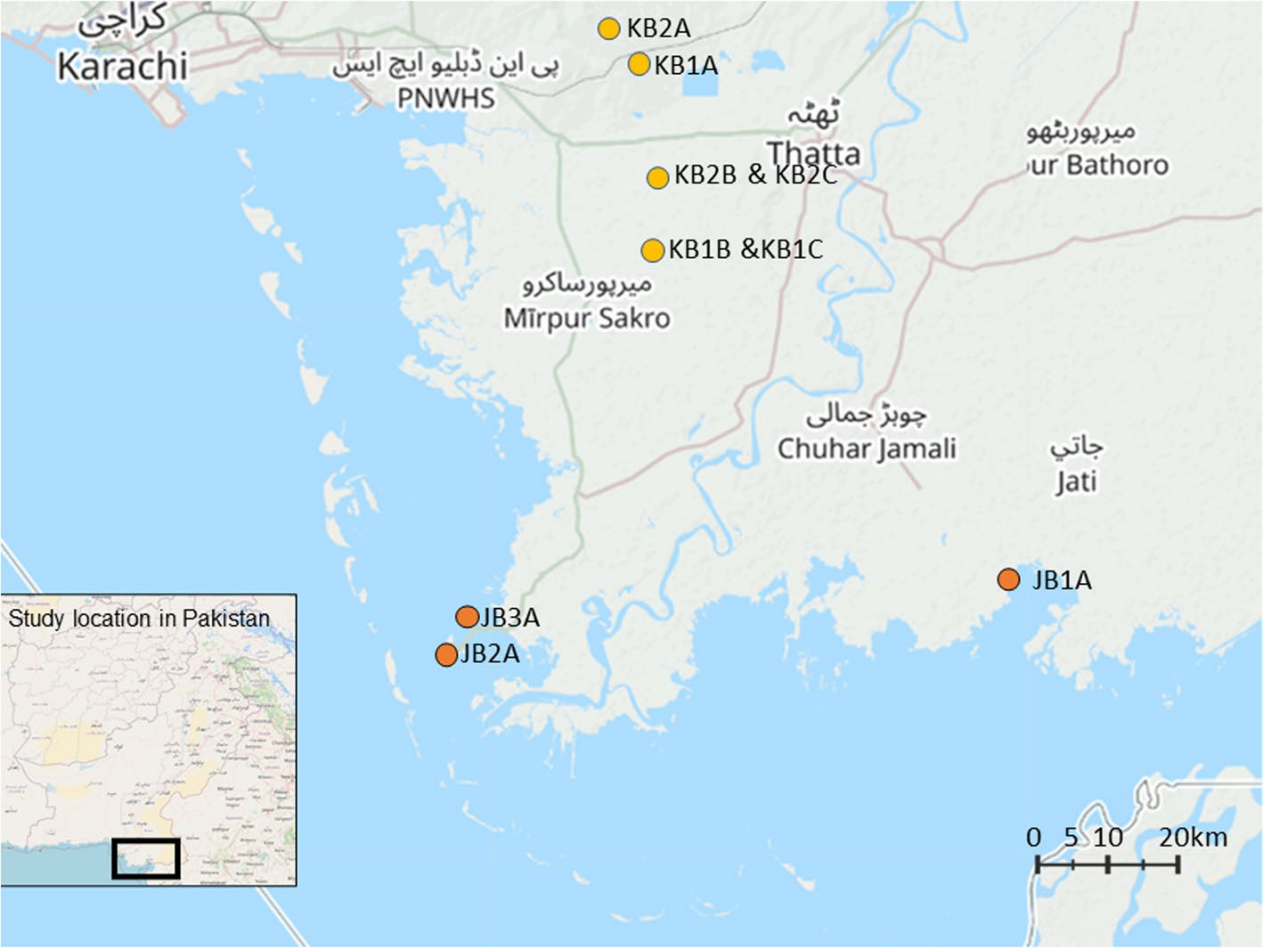
Approximate fieldwork locations (adapted from Open Street Map)

Using purposeful sampling methods based on displacement status and livestock husbandry,

respondents were identified from the Kalmati and Jat Baloch tribes by local staff members from Sindh’s Planning and Development Department and the World Wide Fund for Nature (WWF) Pakistan, through exploration by car and on foot. The Baloch people live across Afghanistan, Iran and Pakistan, with the majority in Pakistan across Balochistan and Sindh provinces. There are over fifty Baloch tribes, with the Jati and Kalmati two of the tribes settled in the Delta region of the Indus. The communities we visited live in marginal areas, in off-grid, unregistered villages. Coastal communities were visited in Jati and Keti Bandar municipalities, and seasonal migrants in both their home village near Ghulamullah and Jungshai (Fig. 1).

Communities consist of 16 to 75 households, clustered into patrilineal cultural groups. Kalmati Baloch households own between 10 and 50 livestock, usually a mix of buffalo, cattle, sheep and goats, with buffalo considered the most valuable and least susceptive to disease. During the day livestock is herded away from their homes, but at night the animals are tied close by, guarded by young boys against theft. Historically, the Kalmati Baloch used to keep camels as well, however, respondents mentioned these were lost to disease or sold, and currently only Jati Baloch own camels, primarily as status symbol [29].

Historically, camel herders belonging to the Jat clan lived a nomadic lifestyle: for six months the camels were kept in the mangrove areas, and the remaining six months herders moved along with their camels [29]. Although the British colonial authorities offered land ownership rights to the Jat on the condition that they would settle there permanently, this was rejected out of fear that the former owners of the land would ban the grazing of their camels, which is exactly what happened [29]. The camel herders were forced to settle permanently in the eastern part of the Delta, public land with mangroves for camel forage, supported by subsistence fishing (Memon and Thapa, 2011). Over time, fishing became a commercial activity as a result of the increasing demand for fish and fishery products [29].

Small scale fishing remained a productive sector until the early 1980s before the industrialisation of the sector. Currently a variety of vessels and nets are used: as small manual fishing boats are restricted to day fishing in shallow coastal waters, mechanized medium size boats of up to 12 m long have become more common, with fishing trips lasting up to a week [30]. The number of fishing vessels increased from 1,000 manual boats in 1955 to 17,000, of which 4,500 mechanised by 2008, and a tenfold increase in fish landing [11]. Fishing resources are under further pressure by the use of destructive fishing nets with small mesh size, catching juvenile fish, hampering replenishment of the fish stock [29]. Estimates show that up to 70% of fish stocks have been depleted over the last decades [14].

### Data collection and analysis

Respondents (*n* = 35) were purposefully sampled based on displacement status and livestock ownership (buffalo, cattle, goats and/ or camels). Households – defined by the Pakistan Bureau of Statistics as those sharing kitchen facilities – consist of 4–12 members, usually parents and their children. In October and November 2019, semi-structured interviews were conducted with those adult household members responsible for animal husbandry and/ or livestock product processing, both male (*n* = 28) and female (*n* = 7). To counter gender-based barriers to data collection, the fieldwork was supported by both a female and male facilitator/ interpreter. Data collection was conducted in respondents’ indigenous language Sindhi, and translated into English. Interviews were recorded after approval from the respondent, and transcribed into English.

While I set out to conduct individual key informant interviews, the communality of households in Sindh, as well as cultural and individual traditions within families meant that conversations were rarely one-on-one [31], which I defined as Focus Group Discussion (FGD), however adopting the same protocol and research tools (S1). Where I was able to conduct individual conversations, there were no facilities available to conduct these interviews in a separate space, and as a result household members often walked in and out of the conversation.

Observations within and around the household and community were recorded through photos and field notes to support contextualization and triangulation of the responses. Following the qualitative research principle of data saturation, interviews continued until no new data and concepts were introduced, using a flexible approach for applied inductive thematic analyses [32, 33].

Interview and secondary data were coded manually and synthesized into matrices in English, and themes constructed from the data using a thematical analysis approach [34]. The matrices are available upon request to the author. Findings were triangulated with primary and secondary data, consisting of a wider literature review on livestock, public health and diseases in Sindh. The constructed themes were interpreted drawing on the ecosocial theory, using a multidisciplinary approach to analyse individual- and community-level data in ecological and social contexts [35]. The global themes form the structure of the results section, identifying the multilevel factors, dynamic processes and pathways affecting zoonotic disease risk in environmental displacement contexts.

### Theory

The multifaceted factors determining health have been described by WHO as the ‘social determinants of health’: the non-medical factors determining health outcomes, including socio-economic, living conditions, food insecurity, conflict and access to (health) services [36]. Similarly, studies into zoonotic disease emergence and transmission increasingly use inclusive frameworks such as One Health, Ecohealth and Planetary Health to improve the consideration of linkages between veterinary, human, and ecosystem health [37]. While these new approaches are valuable in terms of describing health outcomes within environmental and planetary boundaries, especially in light of climate change, these rarely focus on the underlying mechanisms creating health inequalities, or the ‘determination’ rather than the determinants of health [38].

Krieger [39] argues that non-contextual description of social determinants hampers understanding of causes of disease. Rather than focusing on the negative health outcomes of displacement, such as overcrowding and unhygienic living conditions, these can be framed as biological expressions of pre-existing and structural inequalities, rooted in the historical, political and socio-economic context [39]. Krieger developed the ecosocial theory to describe the physical embodiment of biological, environmental, political and economic processes, including socio-economic inequalities [35], which is highly relevant in often structurally marginalised displaced populations. With global historical and political processes having caused enormous inequalities in the responsibility for ecosystem changes, and the resulting loss and damages in poorer countries and communities, the ecosocial theory can be applied to improve understanding of epidemiological risk factors, by accounting for the unequal burden of disease on the poor.

### Ethics

The study protocol was approved by the Human Biology Ethics Committee at the University of Cambridge (protocol number HBREC.2019.25) and in Sindh by the Planning and Development Department, Research and Training Wing of the Government of Sindh. Information and informed consent letters were available in English and Sindhi. Participants were informed about the study during face-to-face recruitment, and informed about the voluntary bases of participation at the start of data collection. Copies of the study protocol and consent forms were available, and all participants provided verbal consent for data collection and recording of interviews.

The power imbalance between researcher and participant was mitigated as much as possible by the selection of households and individual participants through local members of the respondents’ communities, while both male and female interpreters ensured access to all livestock keepers in the households. Asked about gendered roles within the communities, some respondents were hesitant to share their viewpoints, anticipating a negative impression by the foreign researcher, which was mitigated through translation and transcription. In particular among the Jati Baloch, I did not get permission to speak at length with women, however, these were visited during observational studies, and they provided responses to unstructured questions. Among the Kalmati Baloch, which shared their tribal affiliation with our male facilitator, I was able to talk to women separately through a female interpreter and repeated visits to build trust.

## Results and discussion

During October and November 2019, interviews were conducted with 35 respondents, with conversations lasting up to two hours. Respondents were adult members of the Kalmati Baloch (*n* = 13, of which 7 women) and Jati Baloch (*n* = 22, all male) clans (Table 1).

**Table 1:** Respondents’ characteristics

Community (code)	Number of respondents	Gender	Location (closest town)	Displacement (estimate)
Kalmati Baloch (KB1a)	2	Male/ female	Jungshai	25 km during monsoon
Kalmati Baloch (KB2a)	3	Male	Jungshai	18 km during monsoon
Jati Baloch (JB1a)	6	Male	Jati	Since 1987
Jati Baloch (JB2a)	9	Male	Keti Bandar	Since 1999
Jati Baloch (JB3a)	7	Male	Keti Bandar	Since 1960
Kalmati Baloch (KB2b)	4	Female	Mirpur Sakro	Returnees
Kalmati Baloch (KB2c)	1	Male	Mirpur Sakro	Returnees
Kalmati Baloch (KB1b)	2	Female	Ghulamullah	Returnees
Kalmati Baloch (KB1c)	1	Male	Ghulamullah	Returnees
**TOTAL**	35			

The themes identified throughout the interviews were: community, location, environment, livelihood, nutrition, services and support, disaster, displacement, health and disease, animal and veterinary health. Respondents discussed these in response to the semi-structured interview questions, which served both to understand the displacement context and potential areas of zoonotic disease risk and vulnerability. Zoonotic disease dynamics are influenced by complex and interlinked drivers; identified factors for zoonotic disease risks include environmental changes, including through disaster, socio-economic profiles, living conditions and access to (veterinary) health services [9]

The Jati Baloch communities settled on public ‘waste’ (JB2a) land between 1960 and 2000, moving inland as a result of sea intrusion and coastal erosion. Ongoing environmental degradation continues to threaten their current locations, while heavy monsoon rains and cyclones regularly cause floods and an increase in mosquitoes. A cyclone in 1999 was particularly destructive, when over 3,700 villages and 1158 km2 of agricultural crops were destroyed [11]. Floods up to four feet high killed two people and half of all livestock in one respondents’ community. Others recollected losing homes and possessions as the dam breached, saving their children on their fishing boats. Their camels were left to fend for themselves, as “camels are safe, they can swim” (JB3a). These Kharai, or ‘swimming camels’, are slowly becoming extinct due to the destruction of their ecosystem [40]. While the loss of animals may reduce the presence of zoonotic pathogens, this also reduces the availability of food and nutrition, impacting people’s health and in turn their immunity against zoonotic pathogens from other sources, including as a result of environmental changes.

The Kalmati Baloch communities move annually during the monsoon in the late summer months, while slow-onset disasters such as drought and salination further impact their livelihoods. Households move independently over 18 – 25 km, and may choose different destination locations than other community-, or even family members, depending on the availability of food and water for themselves and their livestock, as available forage does not suit all animals. While this may improve nutrition and animal health, in these areas animal and human populations interact with those from other villages, which may increase their vulnerability to zoonotic pathogens they were not earlier exposed to.

One or two older, less productive animals are sold to rent vehicles to move women and young children, while the rest of livestock are herded by adult and adolescent men. As resources are scarce in the drier hilly areas, utensils, charpais (beds) and shelter material, consisting of wooden sticks and fabric, are brought from the origin village, while an open space lined with large stones shaped in a circle acts as a praying area in lieu of a mosque.

"How could someone find wood in this area (…) there is not a single tree" (KB1A, Kalmati Baloch male, Jungshai)

There are distinct tribal and cultural differences between the Kalmati and Jati Baloch communities, as well as within families and households, reflected in livelihoods and displacement context, gendered roles and livestock keeping, which influences which specific zoonoses people are vulnerable to. Furthermore, men and women experience displacement differently, as women are more involved in preparation and restoration, while also responsible for taking care of sick household members and animals, increasing their risk of zoonotic disease.

Below findings are presented based on the main themes constructed from the thematic analysis, drawing on the differences and similarities between respondents to support recommendations for further research and policy.

### When the water comes

Both abundance and shortage of water is linked to ill health and disease in the literature. Water-borne zoonoses are common in flood related disasters [41, 42]. Respondents only moved once water physically entered their lives, destroying homes and agricultural land, however, disease itself is an important secondary driver for displacement. Animal and human disease determined respondents’ displacement dynamic, moving to avoid disease, or becoming restricted by disease outbreaks.

Among the Kalmati, each year animals are lost through injuries, the increase in vectors, or through infectious diseases in flooded areas or at the destination location, as floods exacerbate the burden of insects, pests and pathogens in the Delta’s warm and humid environment [16]. One man and his animals died during the 2019 monsoon as a result of a traffic accident, while snake bites are common.

"Two of our calves died [a week] after coming here, I have no idea what disease they got, they [were] drenched in water, the place was flooded with rain water when we left (…) My cousin lost his cow and [another] cousin lost two or three sheep." (KB1a, Kalmati Baloch male livestock herder, Jungshai).

Dead animals are buried, as respondents know from experience the risk of infectious disease spread. The Jati Baloch and their camels experienced fever and cough in the aftermath of the 1999 floods, while camels suffered infertility due to malnutrition following the disaster. Exhaustion and a lack of nutrition as a result of the disaster or displacement are other causes for livestock loss, affecting households’ food security.

Respondents’ livestock is regularly affected by the non-zoonotic, but highly contagious Foot and Mouth Disease (FMD), locally known as ‘Samarro’, identified through its clinical signs, primarily a loss of milk production, fever, drooling and lesions around the mouth and on the feet, and the loss of young animals [43]. Respondents noted that their animals are particularly vulnerable to the disease in the dry hilly areas, where they graze close to herds from other households and villages.

"If this disease occurs in a nearby village [and] animals roam around and (…) graze together, then our animals can also get this disease" (KB1a, Kalmati Baloch male livestock herder, Jungshai)

Around the time of movement, livestock keepers are particularly careful that their animals do not become infected, as this would restrict their movement for up to three weeks, until the animals have recovered. As respondents cannot afford to lose a sick animal, they invest in veterinary treatment, as “losing an animal is like losing a family member” (KB1b). Respondents are wary of public veterinary and health services however, and private veterinary and healthcare are sought, while prescribed medication is often mixed with traditional methods, such as burning herbs for their smoke to “cure the fever and mouth disease” (KB1a). Although healthcare in larger towns, and especially Karachi was considered better quality, respondents faced difficulties accessing healthcare, due to a lack of female health professionals and language skills, as many health professionals in the province do not speak Sindhi. With veterinary care being much more expensive than human healthcare, only one respondent mentioned buying vaccinations against FMD in the past, no longer having funds available for disease prevention. Milk is so precious that even milk from sick animals is used, posing potential zoonotic disease risk, which may explain the high prevalence of brucellosis in the region, which is mitigated by boiling for preservation purposes when possible.

Having escaped floods, the lack of sufficient quality water in drier areas is a health risk to both animals and humans during displacement. While a water pump was once installed by the Saudi owner of a falconry farm in the displacement location, its mechanical parts were taken away, and currently the community and their animals depend on surface water for drinking and washing. After political developments stopped the regular migration of Jati Baloch in search of water, they now depend on water trucking of brackish soiled water sourced only a few kilometers inland. Water is never boiled however, reflecting households’ lack of time and fuel, all increasing the risk of zoonotic disease transmission.

### Community and livelihood disruption

Displacement, disease and the loss of animals severely disrupted people’s livelihoods and community. The Jati Baloch initially moved separately, based on personal networks and connections, before regrouping in their final destination. Some male youth migrated onwards independently to urban centers such as Karachi to find work. Kalmati Baloch communities and families are similarly divided between rural and urban centers, as the land they own is not enough for subsistence. During displacement this division deepens. While there is a strong ancestral connection to the land in both origin and destination location, households primarily determine their destination location based on the area best suitable for their own livestock. In the hilly areas, communities can count up to 100–300 households, each originating from much smaller origin villages, which increases the risk of zoonotic pathogen spillover between animals, as well as animals and humans.

Socio-economic status is a major determinant of health, with those with fewer resources at increased risk of disease, including zoonoses [44]. As poor households depend on each other, the division of communities and households negatively affects mutual assistance and solidarity, increasing the risk of zoonotic disease.

“we borrow from each other, if someone has money, he gives to the ones who do not (…) we unite in difficult times" (KB1a, male livestock keeper, Kalmati Baloch, Jungshai)

Access to services during displacement are scarce. None of the respondents is connected to the electricity grid, although some households bought small solar home system on loan or through NGOs, however, there is no funding available for maintenance. This impacts the available time to cook and boil milk to eliminate zoonotic pathogens to limit the risk of diseases such as brucellosis. Some households have mobile phones, but the lack of reliable electricity supply means that these are used infrequently, which hampers access to mobile veterinary services, including vaccinations and treatment of potentially zoonotic diseases. Worryingly, none of the displaced children receives any education, which is the bases for improving socio-economic status, knowledge of diseases, and through this overall health status. Kalmati Baloch elders received some education, however, financial difficulties forced them to move to a remote village in Ghulamullah, where their children do not have the ability to go to school. In another Kalmati Baloch village, access to school for children from surrounding villages was blocked by the headmaster, as retaliation for a political conflict over the construction of a road to his land. While Jati Baloch elders also received up to four years of education, nearby schools in the Delta were closed eight years ago due to a lack of teachers.

Livelihoods are disrupted as floods destroy houses and crops, impacting protective shelter, and human food and animal feed supply, negatively affecting immunity against zoonotic disease. Even in regular times there is a lack of food, and “men have to leave early in the morning for their daily work with only one cup of tea to start the day” (KB2b). Owning only a few acres of agricultural land per household, daily wage labour is common among Kalmati Baloch, both in origin and destination locations, shifting from agriculture to construction labour when the fields become flooded. Even temporary displacement can last for up to three months, when no planting can take place, and livelihoods are further disrupted. Additionally, researchers found that the onset of monsoon rains has shifted forwards, with implications for the cropping calendar of rainfed agriculture [45].

Animals are an important asset to the Kalmati Baloch, providing nutrition, food security, and even acting as collateral: "(if) someone sees animals standing at our home, they understand that [we] will return his money even if [we need to] sell [our] animals” (KB1a). They own small to medium size herds, consisting of 10–50 goats, cattle, and buffalo, with a generational shift towards cattle as these are considered ‘healthier’, although these are not highly productive: “a cow produces six to eight liters milk [a day], if they [would] receive extra feed, they could produce 16 L a day”(KB2a). Adolescent boys herd the animals during the day, while women take care of the livestock at home. Some cattle is owned in ‘partnership’, with the primary owner receiving a profit when the animal is sold, while other cows are rented by dairy farms in Karachi during their productive months. Animals are sold after 9–10 pregnancies, at a time when they still produce milk, to fetch the best price in the market, with some money reinvested in a cheaper, younger animal. Younger animals are only sold when “our life depends on [it]” (KB1a). The loss of animals cannot always be offset: one elderly female shared that two decades ago the household owned 20–25 buffalo, which were lost because of a “disease in their throat” (KB1b), with insufficient assets to restock. The lack of nutritious food affects immunity to zoonoses, while together these factors increasingly negatively affect socio-economic status, and therefore health.

The Jati Baloch have stuck to camel keeping mainly for their monetary value and as status symbol [29]. Mangroves and the sea breeze are considered healthy for the camels,recently however, one Jati Baloch community lost about a dozen camels to a disease “from elsewhere through the air” (JB3a), after consulting veterinarians and self-administering medication. The last time that so many camels succumbed to a disease was over thirty years ago, a time when Kalmati Baloch shifted away from camel herding.

"our ancestors used to have camels as per our Baloch tradition (…) My maternal grandfather used to have 500 goats (…) and I too used to have goats (…) but then some diseases spread among them (…) three of them died in just one night and then I sold out the [other] one as well [and] I brought a gold nose pin for my wife out of that money" (male livestock herder, Kalmati Baloch).

Adolescent boys herd the camels on remote islands for over half the year, and the camel milk therefore only has a limited impact on a household’s nutrition and health status. As it takes half a day to reach these islands by boat, the boys cannot go to school, and sometimes even lack fresh water supply as bad weather or the price of fuel prevents supplies from reaching the island. The limited fuel available is primarily used for fishing, an occupation the Jati Baloch depend on since their permanent displacement, as agricultural land and livestock was lost, and while this has led to a reduced risk of zoonoses from livestock, respondents still consider this loss overall detrimental to food security, nutrition and health.

In the past two decades, around 7% of farmers in the Delta have abolished farming, with wider implications for the region and country’s food security [46]. The decision to change livelihood or exit farming altogether is not an easy or straightforward one, instead depending on a combination of high input and low output prices, water shortages, and weather shocks [46]. Respondents blamed upstream irrigation for their losses and deteriorating living conditions. Similarly, Jati Baloch blame salinisation on the lack of Indus river water, however, while they see the sea approaching, they are not preparing to leave again, as their “forefathers were buried here” (JB1a), and fearing the loss of their livelihood.

‘we are safe when we are near the river, if we leave the river there is no livelihood for us’ (JB2a, Jati Baloch male)

Prior to extensive waterworks upstream, the port city of Keti Bunder was reportedly a 'cosmopolitan coastal community', providing financial support to other cities, including Karachi [14]. In the surrounding area, livestock husbandry, agriculture and forestry were primary livelihoods, with agricultural produce slowly shifting from rice to sugarcane to tomatoes, as fresh water became increasingly scarce [14], impacting health status of both animals and humans, as well as contributing to deforestation and the loss of forage for animals. As a result of environmental degradation and increasing levels of intergenerational poverty, people migrated or reverted to fishing [14]. Fishing requires significant and recurring investment in the maintenance of boats, fishing nets and other equipment however, forcing many fishers to obtain loans, which are difficult to repay as profits are limited by commissions charged by middlemen and auctioneers [14]. Karrar describes fishing therefore as a 'credit driven industry with endless cycles of borrowing and repayment', in itself a form of feudalism similar to the landholding system common across the rest of the province [14], and causing a deterioration in people’s socio-economic status, thereby in turn impacting health [9].

As people diverted livelihoods to fishing, the mangroves briefly benefited from a reduction in camel grazing, however, the increase in marine fishing now threatens the sustainability of fishing resources instead [29], impacting food supply, nutrition and immunity against disease. The communities witnessed a decline in fish stocks, with respondents complaining that some fishers continue to use small mash nets even though these are illegal, while respondents themselves are unable to fish at deep sea as their boats are too small. Disasters keep having significant impact on their livelihoods, and during our interviews, respondents were not allowed to go fishing following a cyclone alert by the Pakistan Maritime Security Agency (PMSA), losing much needed income.

### The poverty spiral and impact on disease

"Difficulties… this world is full of difficulties. When it rained heavily [the village] became so dirty and full of mosquitoes [threatening] our children and animals (…) then when we came here we faced great problems too (…)" (KB1a, male livestock keeper, Kalmati Baloch).

Each displacement cycle, whether permanently inland or seasonal temporary movement, disrupts communities and livelihoods, increasing precariousness, and susceptibility to zoonotic disease. Zoonotic disease risk is dynamic however, for instance as livestock is lost to accidents, disease or sold to afford displacement, zoonotic disease risk decreases. Meanwhile the loss of assets and nutrition increases the risk to animal and human health, in particular when healthy adult livestock is replaced with new animals of unknown health status. Furthermore, losing livestock limits the ability to recover, with households spiraling further into poverty following each displacement.

Social capital in the district depends on 'the canal water availability, the drainage system availability, land holding, the farming experience and the family size' [47]. Unsurprisingly therefore, the little socio-economic data available show that poverty among communities in the Delta is increasing due to environmental changes and degradation [15, 29], with severe impact on health status. While the social determinants of health explain disease as a result of individual socio-economic risk factors, the ecosocial theory reflects better the situation in the Indus Delta, where structural inequality is embodied by ill health of both animals and humans. As people are getting poorer facing extreme hardship, they are stuck in a downward health spiral. Skin, respiratory, gastrointestinal problems and fever, including malaria, are considered ‘normal’ seasonal diseases:

"By the grace of God, everyone is almost fine. But things like fever are normal" (KB1a, Kalmati Baloch male livestock keeper)

One adolescent boy returned to his village after contracting typhoid fever while working in a garment factory in Karachi, with an outbreak of extensively drug-resistant (XDR) typhoid fever ongoing across Sindh since 2016 [48]. After the initial medication did not cure the disease, there is no more money to continue treatment. Furthermore, infant mortality is high, with one respondent losing five out of their seven children. While the determinants of health describe the influence on health and disease as a result of a lack of healthcare access, lack of water/ nutrition, and substandard living conditions, the process which determines these risks is rooted in political factors.

While environmental displacement impacts health status, environmental degradation and the capacity to respond are rooted in political decisions, both at national and international level, as highly developed countries are disproportionally responsible for global pollution and climate change related impacts. In climate change policy development, the concept of 'climate suffering' is linked to loss and damage in an attempt to support mitigation and adaptation, including through migration [26]. In this context, 'climate trauma' can be a direct result of disasters as well as being displaced.

"it is always the poor who suffer from floods" (KB2b Kalmati Baloch, female livestock keeper)

Due to the localised economy of the Indus Delta [14], there seems to be little political interest in improving the lives of the coastal population, while the lack of coordination between overlapping and competing institutions mandated for coastal management hinders the development of sustainable policies and responses [30]. Inequality rooted in colonialism has been exacerbated by structural adjustment programs (SAPs) forced onto Pakistan by international financial institutions, which continue to claim that the most efficient allocation of resources is one that maximises economic returns, even when faced with the detrimental impact of economic growth on climate change. SAPs have resulted in inadequate freshwater flows allocated to downstream ecosystems, which are considered as unproductive compared to irrigated agriculture [11]. Furthermore, social healthcare practices were lost due to the enforced privatisation of public health structures. These developments have negative consequences for people’s risk of zoonotic disease, as these are influenced by the environmental changes as a result of climate change and disaster, the lack of access to (veterinary) healthcare and other services, and low socio-economic status described above.

The reduction of veterinary and public health services have led to an underfunded sector unable to comprehensively monitor and respond to disease [49]. In Sindh, sero-prevalence studies are rare, and primarily conducted in large-scale farming contexts, rather than in areas where people rely on subsistence farming. Zoonotic disease data is often based on proxies such as ‘respiratory illness’ for tuberculosis and ‘dog bites’ are reported as rabies, while few cases are diagnostically confirmed. Data is stored in paper format in the archives of provincial government departments and links between datasets have not been analysed.

Rooted in a feeling of helplessness, respondents were discontent with their situation, location, the government, local health professionals, and deeply distrusted political elites, sometimes resenting those better off. Some respondents only participated in the study because the facilitator and translator belonged to their own tribe. Fishers blame illegal fishing on 'outsiders' who disregard local resources, backed by regional elites chasing after profit [11]. The failure of the 1991 Water Accord is blamed on Punjab province, which is 'viewed with contempt because of its dominant and dominating positions in politics, economy and the military' [17]. Respondents rarely interact with outsiders, only sporadically meeting NGO staff or healthcare professionals: "they come in this area just like you (…) [for instance] some agriculture people [told us which] sort of spray we should use for our crops" (KB1a, male livestock herder, Kalmati Baloch). No government assistance is received, except for the polio drops distributed by Lady Health Workers, a relatively successful scheme inherited from a more socialist era.

"the government does not care about human health, let alone animal health" (KB2b, Kalmati Baloch female)

## Conclusions

Zoonotic disease dynamics change through the disruption of livelihoods and communities, as a result of disasters and displacement. Both zoonotic and non-zoonotic diseases play an important role in communities’ and household decisions around displacement, with people avoiding contaminated water and vectors, while sick animals hampered movement. While respondents were familiar with the concept of infectious disease, their dependence on livestock, fish and their products does not allow for much consideration around zoonotic disease. The lack of access to veterinary and human healthcare in the Delta is another result of structural inequalities, with the lack of access to education and electricity further exacerbating intergenerational ill health outcomes, decreasing awareness of, and ability to prevent to zoonotic disease pathogens.

Social relations and historical processes do not only affect people’s vulnerability to zoonotic disease, but also to disasters and displacement in general. Gendered roles result in women being considerably worse off, as they are constantly preparing, caring and repairing around disaster and displacement, while taking care of sick animals and household members. There is solidarity among relatives and closest community members, however, competition for resources and services was reflected in some of the respondents’ interactions with, and mistrust of outsiders. The precarious conditions of environmental displacement through the disruption of communities and livelihoods makes respondents vulnerable to disease, while their awareness of who is responsible for these environmental conditions, limits their willingness to look for outside support. This is an important challenge to address in light of increased (permanent) displacement in the region and globally, and may affect people’s vulnerability to zoonotic disease as they may be unwilling to access veterinary and public health services.

Displacement itself creates a poverty spiral, as the mechanisms employed by displaced populations exacerbated hardship and lowered people’s socio-economic position, thereby increasing risks to health. Coping mechanisms to displacement include selling livestock to enable movement, which in itself might reduce the risk of zoonoses, while the associated loss of assets, nutrition, and the subsequent acquiring of new young animals with unknown health status, might in fact increase risks. Even in the absence of disasters and displacement, people are already ‘trapped’ in poverty, with predictable displacement worsening health and socio-economic status over time. Decreasing wealth over generations as a result of environmental degradation through political and economic choices both locally and globally are ‘embodied’ in deteriorating health conditions of both animals and humans.

Whether permanently or temporarily displaced, communities all embody the resulting suffering, with health considered only the absence of extraordinary disease. Differences in permanent or temporary displaced respondents’ lives and experiences were linked to their tribal affiliations and ancestry, determining levels of mobility and immobility, destination location, available resources, and the type of livestock kept. Their vulnerability to zoonotic disease and health outcomes were similarly rooted in multilevel structural inequalities.

Environmental degradation in the Delta is caused by upstream diversion of Indus river water, overgrazing and forestry, as a direct result of economic choices by a remote political elite, and resulting in the loss of sustainable livelihood options of the people living in the Delta. The environmental impact of climate change, rooted in global and national political choices, disproportionally impacts poor communities without the resources to prepare or respond to disasters. This uneven responsibility versus loss is increasing in the context of climate change. Climate change and environmental degradation itself can increase the risk of zoonotic disease emergence, through biodiversity loss and increased interaction between wildlife, domestic animals and humans, although evidence from the delta showed a decreasing livestock herd as a result of deforestation, limiting presence of zoonotic pathogens in households. The destructive impact of global and national political decisions on Jati Baloch livelihoods and migration patterns act as a warning to protect other communities in the Delta, all at risk of compound environmental risks and permanent displacement impacting the health of animals and humans.

### Policy and planning implications

Environmental displacement as a result of ecological collapse due to climate change is a growing global challenge, particularly affecting poor marginalised communities. Protracted and recurring environmental displacement is projected to increase, requiring reconsideration and reframing of humanitarian and development policy and implementation to mitigate zoonotic disease risks. Policy and interventions need to provide not only ad-hoc support and displacement ‘solutions’, but better consider far-reaching outcomes of political and economic decisions to poor communities facing a perpetual decline in conditions. Addressing the environmental, social and economic effects of water diversion to prepare for disasters, will help prevent not only displacement, but also zoonotic disease risk factors and impact to health and livelihoods. The feasibility of the below implications and recommendations not only depends on political will, but on detailed contextualization, including of the implications to other areas of communities’ lives. This must be based on inclusive consultations with the affected communities themselves, to ensure viability and ownership.

As respondents are familiar with infectious disease transmission routes, they can play an important role in the early detection and prevention of infectious diseases once properly supported, including zoonoses through their lived experiences of animal and human disease outbreaks in some of the least accessible areas of the Indus Delta. To enhance their agency, communities need to be provided with accessible services, importantly education. At provincial and district level, access to veterinary and human health care needs to be improved, through better infrastructure, increased training and employment of female health practitioners and more inclusive language skills of health professionals.

There is trend towards ‘resilience’ programming among donors and humanitarian responders to bridge the gap between humanitarian aid and development assistance, however, implementation is lacking. Appropriate adaptation and response mechanisms need to be put in place, including options for safe migration for those who are no longer able to stay in an unlivable environment. This includes addressing global inequities between climate change responsibility and loss and damage need to be urgently addressed, an issue highlighted but largely ignored by the most significant polluters during the most recent Conference of Parties (COP26) in Glasgow.

### Limitations

The qualitative case study methodology does not aim to provide generalizable research findings, acknowledging that people and phenomena depend on their context and circumstances, although some of its findings may be applicable to similar groups in comparable situations. While qualitative studies do not necessarily provide statistical relevant samples, the results of this study are relevant to countries and regions facing similar challenges, increasingly relevant in the context of the ongoing climate emergency. Selection bias was introduced by selecting two Jati Baloch communities which received, and continue to receive assistance by WWF, while recall bias may have occurred in those communities where disaster and displacement events were not recent.

## Supplementary Information

**Additional file 1: Supplementary material S1. **Research Tools.

## Data Availability

The datasets used and/or analysed during the current study are available from the corresponding author on reasonable request. Questionnaires are provided as Supplementary material (S1_Research_Tools).

## References

[1] Karesh WB, Dobson A, Lloyd-Smith JO, Lubroth J, Dixon MA, Bennett M (2012). Ecology of zoonoses: natural and unnatural histories. Lancet.

[2] Jones B, McKeever D, Grace, D, Pfeiffer D, Mutua F, Njuki J, et al. Zoonoses (Project 1). Wildlife/domestic livestock interactions. A final report to the UK Department for International Development and ILRI. 2011. https://cgspace.cgiar.org/bitstream/handle/10568/12457/DFID%20FINAL%2025-9-2011.pdf?sequence=1&isAllowed=y. Accessed 29 Dec 2022.

[3] Arif S, Thomson PC, Hernandez-Jover M, McGill DM, Warriach HM, Heller J (2017). Knowledge, attitudes and practices (KAP) relating to brucellosis in smallholder dairy farmers in two provinces in Pakistan. PLoS ONE.

[4] Ahmad W, Naeem MA, Akram Q, Ahmad S, Younus M (2021). Exploring rabies endemicity in Pakistan: major constraints & possible solutions. Acta Trop.

[5] Braam DH, Chandio R, Jephcott FL, Tasker A, Wood JLN (2021). Disaster displacement and zoonotic disease dynamics: the impact of structural and chronic drivers in Sindh, Pakistan. PLOS Glob Public Health.

[6] Scoones I, Jones K, Lo Iacono G, Redding DW, Wilkinson A, Wood JLN (2017). Integrative modelling for one health: pattern, process and participation. Philos Trans R Soc B Biol Sci.

[7] Watson JT, Gayer M, Connolly MA (2007). Epidemics after natural disasters. Emerg Infect Dis.

[8] Galvani AP, May RM (2005). Dimensions of superspreading. Nature.

[9] Braam DH, Jephcott FL, Wood JLN (2021). Identifying the research gap of zoonotic disease in displacement: a systematic review. Glob Health Res Policy.

[10] Nasir SM, Akbar G (2012). Effect of river Indus flow on low riparian ecosystems of Sindh: a review paper. Rec Zool Surv Pak.

[11] Kidwai S, Ahmed W, Tabrez SM, Zhang J, Giosan L, Clift P, et al. The Indus Delta—Catchment, River, Coast, and People. In: Coasts and Estuaries, the future. Elsevier; 2019, p. 213–32. 10.1016/B978-0-12-814003-1.00012-5.

[12] Ministry of Climate Change. National Climate Change Policy of Pakistan 2012. Government of Pakistan. 2012. http://www.gcisc.org.pk/National_Climate_Change_Policy_2012.pdf. Accessed 29 Dec 2022.

[13] Keesing F, Ostfeld RS (2021). Impacts of biodiversity and biodiversity loss on zoonotic diseases. Proc Natl Acad Sci.

[14] Karrar H (2021). The Indus Delta between past and future: precarious livelihoods and neoliberal Imaginaries in a parched coastal belt. J Indian Ocean World Stud.

[15] Mahar GA, Zaigham NA (2021). The impact of environmental changes on indigenous agriculture in the Indus Delta Pakistan: a spatio-temporal assessment. Arab J Geosci.

[16] Rasul G, Mahmood A, Sadiq A, Khan SI (2012). Vulnerability of the Indus Delta to climate change in Pakistan. Pak J Meteorol.

[17] Magsi H, Atif S (2012). The Indus water distribution in Sindh, Pakistan: management, impacts and conflicts. Agric J.

[18] Solangi GS, Siyal AA, Siyal P (2019). Analysis of Indus Delta groundwater and surface water suitability for domestic and irrigation purposes. Civ Eng J.

[19] Kanwal S, Ding X, Sajjad M, Abbas S (2019). Three decades of coastal changes in Sindh, Pakistan (1989–2018): a geospatial assessment. Remote Sens.

[20] IDMC (2021). A decade of displacement in the Middle East and North Africa.

[21] IOM. MC/INF/288 - Discussion Note: Migration and the Environment 2007. Ninety-fourth session of the Council. IOM. 2007. https://environmentalmigration.iom.int/sites/g/files/tmzbdl1411/files/MC_INF_288.pdf. Accessed 29 Dec 2022.

[22] Khan A, Walley J, Newell J, Imdad N (2000). Tuberculosis in Pakistan: socio-cultural constraints and opportunities in treatment. Soc Sci Med.

[23] Heslin A, Deckard ND, Oakes R, Montero-Colbert A, Mechler R, Bouwer LM, Schinko T, Surminski S, Linnerooth-Bayer J (2019). Displacement and resettlement: understanding the role of climate change in contemporary migration. Loss damage clim. Change Concepts Methods Policy Options.

[24] Yin RK (2003). Case study research: design and methods.

[25] Ebneyamini S, Sadeghi Moghadam MR (2018). Toward developing a framework for conducting case study research. Int J Qual Methods.

[26] Ajani A, van der Geest K (2021). Climate change in rural Pakistan: evidence and experiences from a people-centered perspective. Sustain Sci.

[27] Haines D (2013). Building the Empire, building the nation: development, legitimacy and hydro-politics in Sind, 1919–1969.

[28] Husain I, Quraishī I, Hussain N (2019). The economy of modern Sindh: opportunities lost and lessons for the future.

[29] Memon JA, Thapa GB (2011). The Indus Irrigation System, Natural Resources, and Community Occupational Quality in the Delta Region of Pakistan. Environ Manage.

[30] Ali MH, Dinshaw RC (2016). A handbook on Pakistan’s coastal and marine resources.

[31] Naveed A, Arnot M (2019). Exploring educational and social inequality through the polyphonic voices of the poor: a *habitus listening guide* for the analysis of family-schooling relations. Comp Educ.

[32] Fusch P, Ness L (2015). Are we there yet? Data saturation in qualitative research. Qual Rep.

[33] Guest G, Namey E, Chen M (2020). A simple method to assess and report thematic saturation in qualitative research. PLoS ONE.

[34] Attride-Stirling J (2001). Thematic networks: an analytic tool for qualitative research. Qual Res.

[35] Krieger N (1994). Epidemiology and the web of causation: has anyone seen the spider?. Soc Sci Med.

[36] WHO. Social determinants of health. https://www.who.int/health-topics/social-determinants-of-health#tab=tab_1. Accessed 29 Dec 2022.

[37] Tasker A, Braam D (2021). Positioning zoonotic disease research in forced migration: a systematic literature review of theoretical frameworks and approaches. PLoS ONE.

[38] Waitzkin H, Winans AP, Anderson M (2020). Social medicine and the coming transformation.

[39] Krieger N (2001). Theories for social epidemiology in the 21st century: an ecosocial perspective. Int J Epidemiol.

[40] Mehta L, Srivastava S, Movik S, Adam HN, D’Souza R, Parthasarathy D (2021). Transformation as praxis: responding to climate change uncertainties in marginal environments in South Asia. Curr Opin Environ Sustain.

[41] Ahern M, Kovats RS, Wilkinson P, Few R, Matthies F (2005). Global health impacts of floods: epidemiologic evidence. Epidemiol Rev.

[42] Baqir M, Sobani ZA, Bhamani A, Bham NS, Abid S, Farook J (2012). Infectious diseases in the aftermath of monsoon flooding in Pakistan. Asian Pac J Trop Biomed.

[43] Ferrari G, Tasciotti L, Khan E, Kiani A (2014). Foot-and-Mouth Disease and its effect on milk yield: an economic analysis on livestock holders in Pakistan. Transbound Emerg Dis.

[44] Dahlgren G, Whitehead M. Policies and strategies to promote social equity in health. Background document to WHO - Strategy paper for Europe. Institute for Futures Studies. 1991. https://core.ac.uk/download/pdf/6472456.pdf. Accessed 29 Dec 2022.

[45] Ali S, Khalid B, Kiani RS, Babar R, Nasir S, Rehman N (2020). Spatio-temporal variability of summer Monsoon Onset over Pakistan. Asia-Pac J Atmospheric Sci.

[46] Ahmad MI, Oxley L, Ma H (2020). What makes farmers exit farming: a case study of Sindh Province Pakistan. Sustain.

[47] Sheikh MJ, Redzuan M, Samah AA, Ahmad N (2016). Identifying sources of social capital among the farmers of the rural Sindh province of Pakistan. Agric Econ Zemědělská Ekon.

[48] Akbani S, Bibi F (2021). A case report of extensively drug resistant typhoid in Karachi, Pakistan: a major Health concern to curb the outbreak. Eur Med J.

[49] Peters LER, Shannon G, Kelman I, Meriläinen E (2022). Toward resourcefulness: pathways for community positive health. Glob Health Promot.

