# Supplementary material for: Zoonoses in the margins: environmental displacement and health outcomes in the Indus Delta
Source: Int J Equity Health. 2022 Dec 30;21:189. doi: 10.1186/s12939-022-01823-0 (PMC9800233; doi:10.1186/s12939-022-01823-0)
Supplement: Supplementary file 1 — Additional file 1: Supplementary material S1. Research Tools. [file 12939_2022_1823_MOESM1_ESM.docx]

**INTERVIEWS: COMMUNITY LEADER**

Objective: to collect information about the households in the community and themes such as poverty, housing and humanitarian assistance.

**1. GENERAL INFORMATION**

1. Can you describe the community?
   1. Prompt: when established (name?); organization village/compound; number of families; education/ facilities; transportation (to where, for what?)
   2. Prompt: tribe, caste, leadership, community roles (tradesman, herder), lineage, marriage, basic unit: household composition
   3. Probe: has the community changed (size/ households) during displacement?
2. What (type/ quantity) livestock is kept by local households?
   1. Prompt: origin, type/ quantity livestock, ownership
   2. Probe: were these livestock owned before displacement? Changes/ why?
   3. Who looks after them/ gender/ how/ where?
3. Can you describe the drought event?
   1. How is the response now different from normal dry times?
   2. Why? Prompt: agricultural practices, kind of feed, water
4. How would you describe how displacement happened here and what effect it has had on people and their lives.
   1. Prompt: Why this location (push/ pull), would you have chosen another location if not a)
   2. Did you move with the entire family? If no where / what do others do?
   3. Can you describe what happened to you and your herd and how it affected your life?
   4. Probe: what animals are used to move? travel measured by daily span, 8-9 miles?
5. Have you been displaced before?
   1. Please describe (as above)
   2. Why do you think it is different this time?

**2. VULNERABILITY AND RISK**

1. What do you think puts people at risk of diseases?
   1. Prompt: which diseases, why (political, environmental, economic, lifestyle, cooking, etc)
   2. Role of livestock in these diseases (case/ slaughter/ preparation)
   3. Probe: Example of last displacement (perceptions, assumptions), actions of communities
2. How much to you think displacement has affected the risk/prevalence of disease?
   1. Probe: has it affected the livestock husbandry? Has that affected humans getting diseases from livestock?
   2. Probe: how much are people worried about these sorts of diseases that might have come from livestock? What precautions might they take? Do they work in your view?
3. Where do you see the responsibility lies for preventing these diseases?
   1. Prompt: Authorities (policies), experts (response, services), farmers (local risk factors)
   2. Probe: what services are available

**3. OCCURRENCE AND PREVENTION**

1. How do you prevent disease?
   1. Is there an area where animals are healthier?
   2. Which animal is associated with good health?
2. Can you tell me about community responses to: zoonoses/ displacement?
   1. Probe: protocol (isolation, restrictions, who cares for?); what was the reason?
   2. Probe: reporting, contact with authorities? Working with households to change their (hygiene) practices?
3. What kind of assistance has the community received from outsiders? Example?
   1. Prompt: type, quantity
   2. Probe: who received what, why?
   3. Probe: are you happy with the assistance provided?
4. I am keen to speak to households in the community that have displaced with/ without livestock, could you identify these and introduce me?
5. Is there anything that you feel I should have asked you or that you want to tell me in relation to this issue?

**INTERVIEWS: HEAD OF HOUSEHOLD (if other than head of community/ herder)**

Objective: to learn about the displacement experience, livestock history, physical resources in household, problems of farming/ risks of health, disease, to determine how vulnerabilities to zoonotic disease transmission risk has changed during displacement.

**1. PERSONAL INFORMATION**

1. Can you describe your household?
   1. Prompt: basic unit/ household composition, roles (daily routine, activities, marketing, shopping)
   2. Prompt: children/ ages; education/ schools (location, level, who attends, why (not); (other) jobs; disability
   3. Probe: emigration, do you have any household/ family/ clan members outside of the community, where are they?
2. Can you tell me/ show me how you live?
   1. Prompt: shelter (#rooms), sheds, hygiene, availability water and food (+source), toilet, bathroom, soap, cooking facilities, electricity, fuel, internet/ phone connection, mosquito net
   2. Probe: how do you build your shelter/ shed, why [perceived risk decrease?]
   3. Probe: can you take me through your daily life
3. Do your children go to school? Did they go to school previously? What kind/ type of school?
   1. How often do your children miss a school day because of health?
4. Do you have enough to eat? What do you eat? Where does it come from? How do you prepare it?
   1. Do you have more or less food now following displacement?
5. Do you own any livestock?
   1. Prompt: origin, type/ quantity livestock; who takes care of these?
   2. Probe: did you own any before displacement
6. What do you do with the animals?
   1. Prompt: transportation, food, bride payment, Eid, offering
7. Household income (other)
   1. Prompt: produce, remittances
   2. Spending: decision making, highest (wedding etc) > lowest
   3. Is there some kind of insurance system? Pooled funds (especially in regular disaster affected communities)

**2. DISPLACEMENT**

1. Can you describe the drought (or flood) event?
   1. How did you respond: change agricultural practices, move grazing, water?
   2. What do you do during normal dry times and how/ why was it different this time?
2. Where did you live previously?
   1. Probe: how did you choose this destination, who made the decision?
3. Can you describe your (displacement) experiences and challenges?
   1. Probe: what animals used to move? travel measured by daily span (8-9 miles)?
   2. Prompt: choice of destination, transport, family members, assets (animals), main issues, insurance
   3. Probe: have you been displaced before? can you describe that experience and whether it was similar/ difference? Return?
   4. Probe: How do you share information about previous experiences? Journal keeping?
4. How has the displacement affected you and your family?
   1. Prompt: resources, environment, shelter, nutrition, income generation of livestock vs non-livestock income sources before and after displacement, spending priorities, animal handling, grazing, slaughter, health
   2. Probe: How do you address these changes? Examples.
5. Can you tell me about your animals and how their situation changed due to the displacement?
   1. Prompt: which animals/ who looks after them (gender) , since when, why these types of animals, shelter (depend on species?), use of animals (changes?), health
   2. Probe how their situation changed due to the displacement/ Did you lose livestock? When and how? How many? death, sales, slaughtered, other?
   3. Probe: have you changed the way you look after them? How – describe husbandry before and now
   4. How did all these things impact your life?

**3. VULNERABILITY AND RISK**

1. How are you feeling today? Do you have any complaints?
   1. How often do you feel like that?
   2. Is there a difference before and after displacement? Concerns, worries?
   3. Did it stop you from working/ school?
2. Can you give me an example of the last time you/ your family member caught a disease?
   1. What caused it? Did you change anything because of this?
   2. What assistance did you seek/ ask for, did you receive it? Relationship to NGOs?
3. When you are ill, do you seek health treatment?
   1. Why (not): location, expense (example)
   2. When do you decide to seek healthcare? Role religion, previous experience, word of mouth, funds (threshold)
   3. Probe: Which specific facilities? [compare with local health data?]
4. Do you use medication, from what source, how much do you spend on these? What resources do you use to pay for treatment/ medication?
   1. Prompt: Income, loan, remittances?
   2. What do you do with any leftover medication? Do you ever use other people's medication?
   3. Do you have any medical kit in the house?
5. Do you or your family members suffer from diarrhea at the moment? How often does this occur? What do you believe causes it? What do you do about it? Did you have these complaints before displacement as well?
   1. Prompt: health/ treatment; medication
6. What do you think puts people at risk of diseases?
   1. Prompt: (political, environmental, economic, lifestyle, slaughter, cooking, etc) risk factors [animals: why do you think that?]
   2. Probe: Example of last displacement (perceptions, assumptions), actions of community

**ANIMAL HEALTH**

1. Which of your animals is most healthy?
   1. Which products are most healthy for your/ families health? Why? What do you do with them/ how do you prepare these?
   2. Is there an animal which poses a risk to your or your children's health?
   3. Have you or family members ever been bitten by a dog? How often did this happen? What did you do when it happened?
   4. Prompt: health/ treatment; medication
2. Can you describe animal illnesses and how you would normally deal with those:
   1. Prompt: severity, symptoms (example)
   2. Probe: Why do you think your animals are more/ less diseased (causes)?

Probe: Help in the community from other households?

1. Prompt: deal with it yourself – Change what you do? how? Separate animals from humans? Split herds? [map]
2. What else do you do with your animals if/ after they are sick (example last time an animal was sick).
   1. Prompt: vaccination, veterinary (or why not?); and if that does not help?
   2. Is there an area where / time when animals are healthier? Why do you think this is?
   3. Probe: Do you report on disease?
   4. Probe: Where do you get your information? How do you know what to do?

NB: If no animals: where are they now and what could have been done so you could keep the animals?

**4. SERVICES**

1. Have you received any [humanitarian] support/ assistance?
   1. Prompt: type, from whom, how do you receive it, is it sufficient/ happy?
   2. Probe: Can you tell me what other services you access and how?

**5. FUTURE**

1. Can you tell me what your plans are?
   1. Prompt: stay here, return, move on?
   2. Probe: what would influence decision?
   3. Prompt: Policies, assistance, services [prevent protracted displacement]
2. What is your main concern in relation to the health in your family? Why is this so important to you?
   1. Probe: what have you done about it?
3. Would it be possible to speak to the household members responsible for herding/ taking care of the livestock?
4. Is there anything that you feel I should have asked you or that you want to tell me in relation to this issue?
5. Are there any issues the interview brought up you would like more guidance on?

With many thanks for your help

**INTERVIEWS: LIVESTOCK HANDLER AND HERDER**

Objective: to learn about their displacement experience, livestock history, physical resources in household, problems of farming/ risks of health, disease etc, to determine how vulnerabilities to zoonotic disease transmission risk has changed during displacement.

**1. PERSONAL INFORMATION (use previous questions if household head is same person)**

1. What does your typical day look like/ daily activities?
   1. Probe: livestock, other chores, where to get water/ feed
   2. Prompt: have these activities changed during displacement?
2. What is your main worry?

**2. DISPLACEMENT**

1. Can you tell me about your (displacement) experiences and challenges? You moved here from quite a long way away, what was that like? How difficult was it?
   1. Prompt: choice of destination, transport, family members, assets (animals), main issues
   2. Probe: have you been displaced before? can you describe that experience and whether it was similar/ difference?
2. How has the displacement affected you and your family? How has moving here affected you? What about your family? Would you say they are happy with the new situation?
   1. Prompt: resources, environment, shelter, nutrition, income generation of livestock vs non-livestock income sources before and after displacement, spending priorities, animal handling, grazing, slaughter, health
   2. Probe: How do you address these changes? Examples.
3. Can you tell me about your animals and how their situation changed due to the displacement? In terms of livestock, what animals did you have before and what you have now? Is it very different? How did it impact your life?
   1. Probe: resources, space, facilities, electricity, water, site, numbers
   2. Probe: which animals do you look after, since when, why these types of animals, where to keep/ shelter (depend on species?), use of animals (changes?), health, births/ deaths
   3. Probe: When and how did you lose livestock?
   4. Prompt: death, sales, slaughtered, other?

**3. VULNERABILITY AND RISK**

1. What is the current status of your/ household members health?
   1. Prompt: zoonotic diseases
   2. Probe: change during displacement, concerns, how is the sickness hurting you?
2. What do you think puts people at risk of diseases?
   1. Prompt: (political, environmental, economic, lifestyle, slaughter, cooking, etc) risk factors [animals]
   2. Probe: Example of last displacement (perceptions, assumptions), actions of community
3. Do you visit health services? Why (not): location, expense
   1. Prompt: When do you decide to seek healthcare? previous experience, word of mouth, funds (threshold)
   2. Probe: Which specific facilities? [compare with local health data?]
   3. Probe: Do you use medication, from what source, how much do you spend on these?
4. Can you describe animal illnesses and how do these impact the household?
   1. Prompt: severity, symptoms
   2. Probe: Why do you think the animals are more/ less deceased (causes)?
5. What do you do with your animals if/ after they are sick (example last time an animal was sick).
   1. Prompt: vaccination, veterinary (or why not?); and if that does not help?
   2. Probe: Do you report on disease?
   3. Probe: Where do you get your information? How do you know what to do?

NB: If no animals: where are they and what could have been done so you could keep the animals?

**4. SERVICES**

1. Have you received any [humanitarian] assistance? What sort of help have you received? From whom? Who helped the most?
   1. Prompt: type, from whom (including solidarity); sufficient?
   2. Probe: Can you tell me what other services you access and how?

**5. FUTURE**

1. Can you tell me what your personal/ family plans are? What will you do in the future?
   1. Prompt: stay here, return, move on?
   2. Probe: what would influence decision?
   3. Prompt: Policies, assistance, services [prevent protracted displacement]
2. Is there anything that you feel I should have asked you or that you want to tell me in relation to this issue?

With many thanks for your help
